# Effectiveness of a 6-Month Lifestyle Intervention on Diet, Physical Activity, Quality of Life, and Markers of Cardiometabolic Health in Women with PCOS and Obesity and Non-PCOS Obese Controls: One Size Fits All?

**DOI:** 10.3390/nu13103425

**Published:** 2021-09-28

**Authors:** Zheng Wang, Henk Groen, Astrid E. P. Cantineau, Tessa M. van Elten, Matty D. A. Karsten, Anne M. van Oers, Ben W. J. Mol, Tessa J. Roseboom, Annemieke Hoek

**Affiliations:** 1 Department of Obstetrics and Gynecology, University of Groningen, University Medical Centre Groningen, 9700 RB Groningen, The Netherlands; wangzhengmedicine@gmail.com (Z.W.); a.e.p.cantineau@umcg.nl (A.E.P.C.); a.m.van.oers@umcg.nl (A.M.v.O.); 2 Department of Epidemiology, University of Groningen, University Medical Centre Groningen, 9700 RB Groningen, The Netherlands; h.groen01@umcg.nl; 3 Department of Public and Occupational Health, Amsterdam UMC, Vrije Universiteit Amsterdam, De Boelelaan 1117, 1105 AZ Amsterdam, The Netherlands; tessavanelten@gmail.com; 4 Department of Clinical Epidemiology, Biostatistics and Bioinformatics, Amsterdam UMC, University of Amsterdam, Meibergdreef 9, 1105 AZ Amsterdam, The Netherlands; m.d.a.karsten@umcg.nl (M.D.A.K.); t.j.roseboom@amsterdamumc.nl (T.J.R.); 5 Department of Obstetrics and Gynecology, Amsterdam UMC, University of Amsterdam, Meibergdreef 9, 1105 AZ Amsterdam, The Netherlands; 6 Amsterdam Public Health Research Institute, 1105 AZ Amsterdam, The Netherlands; 7 Amsterdam Reproduction and Development, 1105 AZ Amsterdam, The Netherlands; 8 Department of Obstetrics and Gynecology, Monash University, Melbourne 3800, Australia; ben.mol@monash.edu

**Keywords:** PCOS, obesity, lifestyle intervention, dietary intake, physical activity, cardiometabolic health, quality of life

## Abstract

Little is known about the difference in effectiveness of lifestyle intervention between women with PCOS and non-PCOS women. In a post hoc longitudinal analysis of a randomized, controlled trial, we aimed to investigate whether infertile women with PCOS and obesity (*N* = 87) responded differently to a 6-month lifestyle intervention program than infertile non-PCOS obese controls (*N* = 172). We evaluated several aspects of the intervention such as changes in diet, physical activity, and dropout rate, as well as the effect on weight, quality of life (QoL), and cardiometabolic outcomes. Multilevel analyses were used, and analyses were adjusted for baseline characteristics such as age, education, and smoking. Although BMI in both groups significantly decreased at 3 months and 6 months, there were no significant differences between the groups at 3 months (adjusted B: −0.3, 95% CI: −0.9 to 0.3, *p* = 0.35) and 6 months (adjusted B: 0.5, 95% CI: −0.4 to 1.4, *p* = 0.29). Women with PCOS and non-PCOS women had similar compliance with the lifestyle intervention in terms of actual change in diet and physical activity. Mental QoL scores were not different at either 3 or 6 months. Physical QoL scores were lower in women with PCOS compared with non-PCOS women at 3 months (adjusted B: −2.4, 95% CI: −4.8 to −0.06, *p* = 0.045) but not at 6 months. Cardiometabolic parameters did not differ between the groups. Our results showed that infertile women with PCOS and obesity and non-PCOS obese controls responded largely similarly to our lifestyle intervention and achieved the same level of improvement in markers of cardiometabolic health.

## 1. Introduction

Polycystic ovary syndrome (PCOS) is a complex endocrine and metabolic condition in women of reproductive age, characterized by chronic anovulation, hyperandrogenemia, and polycystic ovaries, which may result in hirsutism, irregular menstrual cycles, and infertility [1]. In women with PCOS and infertility due to anovulatory cycles, ovulation induction is the first-line treatment [2]. The recent international evidence-based guideline on PCOS [2] emphasized the significant role of diet and exercise in the management of women with PCOS, particularly in those with excess weight, as up to 60% of the women with PCOS are overweight or obese [3]. Lifestyle interventions leading to 5–10% weight loss may reverse anovulatory status, thereby increasing natural conception rates [1,4]. Moderate weight loss may also improve insulin resistance, dyslipidemia, and androgen excess in PCOS [5].

In a recent systematic review, the efficacy of weight management interventions in terms of weight loss and metabolic measures in women with and without PCOS did not differ significantly [6]. This review, however, identified a paucity of high-quality evidence on this topic. Included studies were generally small with a moderate to high risk of bias, with significant dropout rate, which might lead to overestimation of the outcomes. The authors advised that more rigorous research is needed [6]. Moreover, the analysis did not address differences in diet and physical activity between the groups [7]. In addition, quality of life (QoL) is another factor to be considered during lifestyle intervention studies since poor QoL is associated with attrition during weight-reduction programs [8]. In women with PCOS, poor QoL may particularly be expressed as mental problems and depression [9]. It is not clear whether women with PCOS and obesity experience a different QoL during and after lifestyle interventions than non-PCOS obese controls.

We previously performed a randomized, controlled trial (RCT) to investigate the effects of lifestyle intervention on reproductive outcomes in women with obesity and infertility: the LIFEstyle study [10]. The intervention effect in this RCT on lifestyle, QoL, and cardiometabolic outcomes showed that women in the intervention arm improved lifestyle and physical QoL scores, as well as decreased their risk of metabolic syndrome [11,12]. To explore whether this effect would be different for women with PCOS, we performed a post hoc analysis within the lifestyle intervention arm of the RCT. We compared lifestyle changes, i.e., diet and physical activity, dropout rate, weight loss, QoL, and cardiometabolic outcomes between infertile women with PCOS and obesity and infertile non-PCOS obese controls during and immediately after the 6-month intervention program of the LIFEstyle study.

## 2. Materials and Methods

### 2.1. Study Participants

This was a post hoc longitudinal analysis of the LIFEstyle study [10]. The LIFEstyle study was a multicenter RCT conducting in accordance with the guidelines set forth in the Declaration of Helsinki in women with obesity and infertility. The LIFEstyle trial was registered in the Netherlands Trial Registry (NTR1530) and approved by the Institutional Review Board of the University Medical Center Groningen (no.: 2008/284). The protocol and main results were published [10,13]. In the original RCT, between 2009 and 2012, a total of 577 women of childbearing age were randomly assigned to a 6-month lifestyle intervention followed by 18 months of infertility treatment or 24 months of infertility treatment. Infertile women between 18 and 39 years old with a BMI ≥ 29 were included, and women with severe endometriosis, WHO III anovulation, endocrinopathies, and previous history of pregnancy-induced complications were excluded from the study. All participants received fertility investigation before randomization. The causes of infertility were divided into unexplained infertility, tubal factor, male factor, anovulation (WHO I; WHO II: PCOS; WHO II: non-PCOS), endometriosis, and cervix factor. 

Participants who were allocated to the intervention arm of the original RCT were included in the current analysis and were divided into two groups according to PCOS status. Participants with missing food frequency questionnaire (FFQ) or Short QUestionnaire to ASsess Health-enhancing physical activity (SQUASH) were excluded (see below). Data collected from pregnant women were excluded from the analyses, since pregnancy influences lifestyle and cardiometabolic parameters. PCOS was considered to be present in case two of the following three criteria were met (Rotterdam criteria [14]): oligo-ovulation or anovulation, clinical manifestations of hyperandrogenism and/or hyperandrogenemia, and ovarian polycystic changes. The PCOS group was compared with the control group, consisting of non-PCOS obese women, irrespective of ovulatory status. 

The lifestyle intervention consisted of caloric restriction and physical activity enhancement combined with motivational counseling. The primary goal of the intervention was to reduce body weight by at least 5% of the original body weight during the 6-month intervention period or to reduce the BMI to below 29. Participants could start infertility treatment once the intervention goal was reached or after the intervention period when no spontaneous pregnancy occurred. 

### 2.2. Diet and Physical Activity

Participants in the intervention arm were asked to consume a healthy diet with decreasing calories by approximately 600 kcal/d compared with their common caloric intake but not less than 1200 kcal/day (Health Council of the Netherlands). Participants could use the Dutch Nutrition Center’s web-based food diary (‘Eetmeter’) to report daily feedback on their food and calorie intake and bring these results for consultations [15]. This web-based food diary was validated for research purposes recently [16]. All participants were asked to report a FFQ at the randomization, 3 and 6 months after randomization. The FFQ contained two sections: standardized questionnaire on food intake and additional frequency and portion size questions. The first part of the FFQ was used for the Public Health Monitor in the Netherlands [17]. This standardized questionnaire consisted of questions about cooking fats, bread, breakfast, vegetables, fruits, and fruit juice. Questions about fruit, juice, and cooked vegetable intake were validated by two 24-h recalls [17]. The second part consisted of questions about snacks, soft drinks and alcoholic beverages, and cream and/or sugar in coffee and tea. We focused on the intake of vegetables (grams/day), fruits (grams/day), sugary drinks (glasses/day), alcoholic beverages (glasses/day), savory snacks (handful/week), and sweet snacks (portion/week). 

In addition to caloric restriction, participants were advised to engage in at least 30 min of moderate physical activities (60–85% of maximum heart rate frequency) two to three times a week. A pedometer (Yamax Digi-Walker SW 200, Develing International^®^, Bunschoten, The Netherlands) was used to monitor daily exercise, aiming at 10,000 steps. The intervention coach recorded the average daily step count during the intervention prior to each consultation. A self-implemented diary was used to record these activities to build awareness and for evaluation during consultations. All participants were asked to complete the SQUASH at the randomization and 3 and 6 months after randomization [18]. Data were collected about commuting, leisure time, household activities, and activities at work or school. The results we focused on were moderate to vigorous physical activity during leisure time (minutes/week), moderate to vigorous physical activity during commuting (minutes/week), total moderate to vigorous physical activity (minutes/week), and number of steps (steps/day).

Of note, the effect of lifestyle intervention on diet and physical activity during and after the study period was published [12,19]. The measurements of diet and physical activity were elaborately described [12,19].

### 2.3. QoL

Participants filled in the 36-Item Short Form Health Survey (SF-36) at the time of randomization and 3 and 6 months after randomization through a web-based questionnaire. The SF-36 is a general health-related QoL measure, consisting of 36 items [20]. This questionnaire consists of a Physical Component Score and a Mental Component Score. A higher score represents a better QoL. The changes of physical and mental QoL scores at 3 and 6 months between original allocated arms was published [11].

### 2.4. Clinical and Laboratory Measurements

During the hospital visits at the randomization, at 3 months, and at 6 months after randomization, body weight in kg, height in cm, waist and hip circumference in cm, and blood pressure in mmHg were measured by research nurses who were blinded to the treatment assignment. Fasting blood samples were collected by venipuncture into a serum and a sodium fluoride vacuum blood collection tube. Samples were kept at room temperature for coagulation, centrifuged at 4 °C, and then stored at −80 °C. Fasting glucose, insulin, triglycerides, total cholesterol, low-density lipoprotein cholesterol (LDL-C), high-density lipoprotein cholesterol (HDL-C), and high-sensitivity C-reactive protein (hs-CRP) were measured. Homeostasis model assessment of insulin resistance (HOMA-IR) was calculated as fasting insulin concentration (μU/mL) multiplied by fasting glucose concentration (mmol/L) divided by 22.5. Subjects were diagnosed as metabolic syndrome if they met more than two of the following criteria: (1) glucose ≥ 5.6 mmol/L; (2) HDL-C < 1.3 mmol/L; (3) triglycerides ≥ 1.7 mmol/L; (4) waist circumference ≥ 88 cm; or (5) blood pressure ≥ 130/85 mmHg [21]. The measurement method as well as intra- and interassay variation of those outcomes were described elaborately [11].

### 2.5. Statistical Analysis

Baseline characteristics were compared between women with PCOS and controls. Data were expressed as mean ± standard deviation or median (interquartile range) for continuous variables and proportions (percentage, %) for categorical variables. Normality testing was performed using histograms and normal probability plots (Q-Q plots) combined with the Kolmogorov–Smirnov (K-S) test. The differences between the two groups were compared with Student’s *t*-test or Mann–Whitney U-test where relevant for continuous variables and the Chi-square test for categorical variables. Dropout rates of women with PCOS and controls were calculated and examined with Chi-square test between the two groups.

We used the generalized estimating equations (GEE) method to analyze longitudinal data, which is preferable over complete-case analysis. We first compared the differences in dietary intake, physical activity, QoL, and cardiometabolic parameters between 3 months and baseline and between 6 months and baseline for women with PCOS and non-PCOS women separately, to investigate the changes over time within the groups. Only “time” (baseline, 3 months, and 6 months) was included in these models as a categorical variable and baseline was used as the reference. Secondly, we analyzed the differences in change over time between women with PCOS and controls. For differences and 95% CI in energy and dietary intake, in steps and physical activity, in QoL, and in cardiometabolic outcomes between women with PCOS and non-PCOS women at 3 and 6 months after intervention, we included “time” (baseline, 3 months, and 6 months), PCOS group (yes/no), and an interaction term between time and PCOS group in the models. The baseline measurement was included as a covariate. We first adjusted models for factors that were statistically significantly different at baseline. Next, we included potential confounders one at a time. If the effect estimate in the models changed more than 10%, we included the variable in the final model. Alcoholic beverages and commuting activities both had a median of zero in combination with a very narrow distribution. Thus, we computed these two variables into categorical variables (yes/no), and corresponding odds ratio (OR) were reported instead of mean difference.

The Statistical Package for Social Science (IBM SPSS, Armonk, NY, USA, version 27.0) was used to perform statistical analyses and GraphPad Prism (San Diego, CA, USA, version 8.0) was used for data visualization. A *p*-value < 0.05 was regarded as statistically significant.

## 3. Results

**Figure 1:**
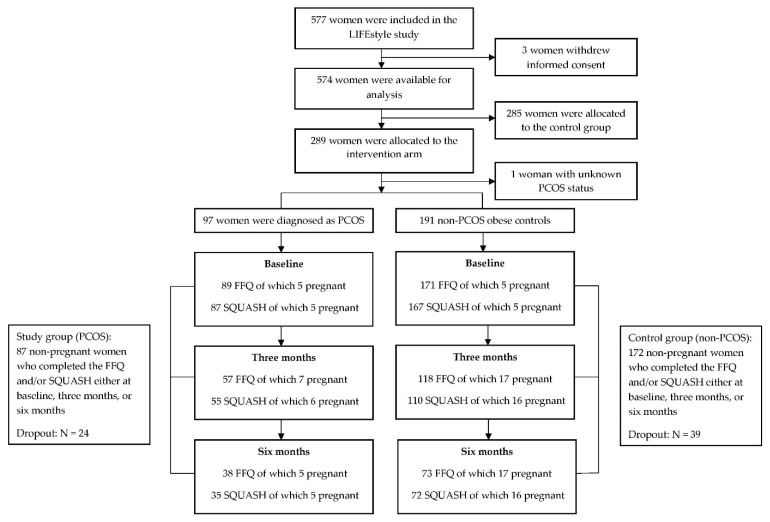
Flow chart of the current study. FFQ: food frequency questionnaire; SQUASH: Short QUestionnaire to ASsess Health-enhancing physical activity.

Of the 577 randomized women, data of 574 women were available for analysis (due to retraction of informed consent). In total, 289 women were allocated to the intervention arm, of whom 97 women were diagnosed as PCOS and 191 women were non-PCOS (one women had unknown ovulatory status). Responders to FFQ and/or SQUASH were decreasing over time due to dropout or failure to respond to questionnaires, resulting in 87 women in the PCOS group, and 172 in the non-PCOS group (28 anovulatory and 144 ovulatory non-PCOS) who completed the FFQ and /or SQUASH either at baseline, 3 months, or 6 months. There were 24 (24.7%) women with PCOS and 39 (20.4%) non-PCOS women who dropped out the intervention, which was not statistically significantly different (OR: 1.28, 95% CI: 0.72 to 2.29, *p* = 0.40). The flow chart of the current analysis is shown in Figure 1. 

Table 1 shows baseline characteristics of included participants in the current analysis. Women with PCOS were significantly younger than non-PCOS women (27.9 ± 3.9 vs. 30.8 ± 4.4 years, *p* < 0.001). Thus, we adjusted for age in all models. The mean BMI was 35.9 ± 3.4 and 36.1 ± 3.4 in the PCOS group and the non-PCOS group (*p* = 0.56). There were no statistically significant differences in ethnicity, education, smoking, and number of nulliparous women. For differences in dietary intake, steps and physical activity, QoL, and cardiometabolic outcomes between women with PCOS and non-PCOS women at 3 and 6 months, models were fully adjusted for education level and smoking based on their impact on the effect estimates together with age and baseline measures.

**Figure 2:**
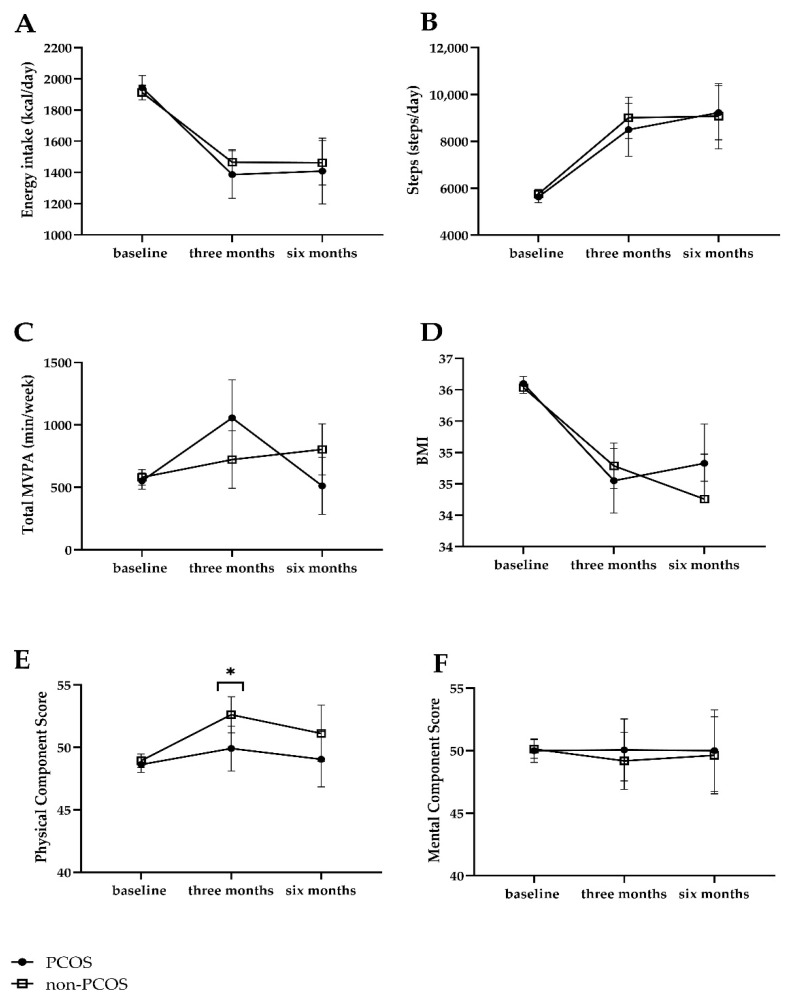
Estimated marginal means of energy intake, steps and total MVPA, QoL, and BMI at baseline, 3 months, and 6 months. (**A**) Energy intake; (**B**) Steps; (**C**) Total MVPA; (**D**) BMI; (**E**) Physical Component Score; (**F**) Mental Component Score. MVPA: moderate to vigorous physical activity. Data were corrected for age, baseline measures, education, smoking at baseline, time, PCOS group, and interaction between time and PCOS group. * *p* < 0.05 between women with PCOS and non-PCOS controls at three months.

Both women with PCOS and non-PCOS women improved lifestyle at 3 and 6 months with respect to energy and dietary intake (Supplementary Table S1). In the comparison over time between women with PCOS and non-PCOS women, they had a similar compliance with the lifestyle intervention in terms of change in lifestyle, i.e., change in energy intake and food groups and change in average steps per day and physical activity (Table 2). Women with PCOS had a higher intake of vegetables (mean difference at 6 months, 24.2 g/day, 95% CI: 1.3 to 47.1, *p* = 0.04) and were more physically active (mean difference at 3 months, 435 min/week, 95% CI: 72 to 798, *p* = 0.02) than non-PCOS women after adjusting for baseline measures and age. However, these statistically significant differences no longer existed in the fully corrected model after adjusting for baseline measures, age, education level, and smoking, although changes in effect sizes were generally small. Estimated marginal means from GEE of energy intake, steps, and total MVPA in women with PCOS and non-PCOS women at baseline, 3 months and 6 months are shown in Figure 2A–C.

With respect to QoL, physical QoL scores were lower in women with PCOS than non-PCOS women at 3 months (adjusted B: −2.4, 95% CI: −4.8 to −0.06, *p* = 0.045), but not at 6 months (adjusted B: −1.7, 95% CI: −4.9 to 1.3, *p* = 0.27). Mental QoL scores did not differ between groups (Table 2). Estimated marginal means from GEE of QoL are shown in Figure 2E,F. 

The body weight or BMI in both groups decreased statistically significantly at 3 months and 6 months, respectively (Supplementary Table S2). There was no difference in BMI between women with PCOS and non-PCOS women at 3 (adjusted B: −0.3, 95% CI: −0.9 to 0.3, *p* = 0.35) and 6 months (adjusted B: 0.5, 95% CI: −0.4 to 1.4, *p* = 0.29). Estimated marginal means of BMI in women with PCOS and non-PCOS women at baseline, 3 months, and 6 months are shown in Figure 2D. With respect to blood pressure, women with PCOS had a lower diastolic blood pressure than non-PCOS women at 6 months (adjusted mean difference: −5.2 mmHg, 95% CI: −10.3 to −0.2, *p* = 0.04) but not at 3 months (Table 3). Markers of cardiometabolic health improved at 3 months and 6 months compared with baseline in both groups, respectively (Supplementary Table S2). Metabolic measures including triglycerides, total cholesterol, HDL-C, LDL-C, hs-CRP, glucose, insulin, and HOMA-IR did not differ between the PCOS and non-PCOS group at either 3 months or 6 months after intervention (Table 3). The prevalence of metabolic syndrome did not differ between the PCOS and non-PCOS group at either 3 months (adjusted OR: 0.59, 95% CI: 0.23 to 1.50, *p* = 0.27) or 6 months (adjusted OR: 0.48, 95% CI: 0.13 to 1.77, *p* = 0.27).

## 4. Discussion

In this post hoc longitudinal analysis, we showed that a 6-month lifestyle intervention aimed at weight loss did not have different effects (i.e., energy and dietary intake, steps and physical activity, dropout rate, weight loss, QoL, and cardiometabolic outcomes) among women with PCOS and obesity and non-PCOS obese controls.

In our previous publication, we showed that all women in our study who followed a 6-month lifestyle intervention decreased their intake of high-calorie snacks and beverages and increased their moderate to vigorous physical activity compared with women without lifestyle intervention [12]. In the current analysis, women with PCOS did not show a different change in lifestyle, i.e., energy and dietary intake, average number of steps per day, and moderate to vigorous physical activity compared with non-PCOS obese controls. Moreover, dropout, which is regarded as a problem in lifestyle interventions, since these reduce effectiveness of interventions in case of high dropout rates [7], was not different between the groups. The rate was in line with a previous literature with a median dropout rate being 24% in overweight and obese infertile women who participate in lifestyle interventions [7]. 

We did not find a significant difference in change of mental QoL scores between infertile women with PCOS and obesity and infertile non-PCOS obese controls during and after lifestyle intervention. It has been argued that lower mental QoL, in general, in women with PCOS compared to non-PCOS women can be influenced by conditions that impair mental QoL, such as infertility and obesity [22]. The literature on change of mental QoL in these groups is scarce, precluding generalizability of our results to fertile women. In addition, we showed that women with PCOS and obesity might have a lower physical QoL score than non-PCOS obese controls at 3 months. The comparison between women with PCOS and non-PCOS women in QoL during and immediately after lifestyle intervention is scarce. Lifestyle interventions have been reported to improve PCOS-specific QoL (growth of visible hair, infertility problems, and feelings of depression) of women with PCOS in a small prospective study (*n* = 49, age: 29 years, BMI: 36) [23]. However, we did not observe statistically significant improvement in physical or mental QoL in women with PCOS over time. We used a general QoL questionnaire (SF-36) in the current study rather than a validated PCOS questionnaire (PCOSQoL) [24], which may have led to different results than the former study [23]. The results of lower physical QoL scores in women with PCOS suggest that this might be a pivot to focus on the physical health of women with PCOS. However, given the relatively small sample size at 3 and 6 months, more studies are needed to verify these findings.

During and after intervention, both women with PCOS and non-PCOS women achieved weight loss, without showing statistically significant differences between the two groups. Both groups showed a better cardiometabolic profile at either 3 or 6 months after the intervention. The only difference over time that we could show was that women with PCOS had a lower diastolic blood pressure at 6 months than non-PCOS women. Again, considering the relatively small sample size at 3 months and 6 months, this might be a chance finding and more studies are needed to show whether our findings are robust.

Of note, the controls in the current study included non-PCOS women regardless of ovulatory status, thus anovulation might be a potential confounder that could have influenced the results. However, due to the relatively small sample size of WHO II anovulation non-PCOS (16% of total non-PCOS), we do not think it would have had a strong effect on the results. Nevertheless, we performed additional analysis between PCOS and ovulatory non-PCOS, and no deviations from the current results occurred.

The main strengths of the current study are the well-characterized study population, the prospective longitudinal nature of the data collection, and the fact that both groups had similarly high BMI. Our findings add to the existing evidence of a previous meta-analysis that showed no statistically significant difference in weight loss between the PCOS and non-PCOS women after lifestyle intervention [6]. Our results constitute important contributions to the scarce evidence and provide potential clinical guidance, i.e., existing lifestyle intervention programs for women with PCOS are feasible and effective in terms of change in lifestyle as well as weight loss and cardiometabolic outcomes. 

However, there are several limitations. First of all, this was not a randomized comparison; we analyzed participants who were allocated to the lifestyle intervention arm of a lifestyle RCT, thus baseline differences existed between the PCOS and non-PCOS women. Although we adjusted for age and baseline measures, residual confounding may have still occurred. The proportion of women who responded to questionnaires or attended hospital visits reduced over the course of the study period due to the nature of RCT with dropouts or due to pregnancies that led to exclusion of further physical examination and blood sampling. Nonetheless, we compared baseline characteristics between women who were included in the current analysis and women who were not included, and no statistically significant differences were found. Second, the use of self-reported questionnaires might lead to underestimation of dietary intake and overestimation of physical activity [25,26]. However, these are presumably not different between the groups since both groups were obese with similar BMI and both groups participated and were coached in the lifestyle intervention. Moreover, we also had data from the step counters to objectify the physical activity of participants; therefore, it is unlikely that this might have influenced the results. Finally, we aimed to exclude women with severe or known eating disorders, but this was not supported by a formal diagnosis and may have led to biased results in our study.

Up to now, there was lack of evidence-based recommendations on what is the best weight loss strategy for women with PCOS and obesity [27]. Given that the results of our analysis showed no statistically significant differences in either compliance with the lifestyle intervention or the effect regarding lifestyle change, dropout rate, weight loss, and cardiometabolic health between women with PCOS and obesity and weight-matched non-PCOS women, current weight management recommendations for the general population could be applied to women with PCOS. At the same time, the results of lower physical QoL scores in women with PCOS compared to non-PCOS controls at 3 months after randomization suggest that additional counseling on physical activity could be important for women with PCOS in future lifestyle intervention studies.

## 5. Conclusions

Our results show that infertile women with PCOS and obesity and infertile non-PCOS obese controls responded largely similarly to our lifestyle intervention, with similar lifestyle changes and weight loss, and achieved the same level of improvement in markers of cardiometabolic health. There is no evidence to handle lifestyle advice to women with PCOS and obesity differently compared to non-PCOS obese women.

## Figures and Tables

**Table 1:** Baseline characteristics of participants allocated to the intervention arm.

	PCOS (*N* = 87)	Non-PCOS (*N* = 172)	*p*-Value
Age (years)	27.9 ± 3.9	30.8 ± 4.4	<0.001
Western European Ethnicity	79 (90.8%)	151 (87.8%)	0.47
Education			0.82
Primary school	3 (3.8%)	10 (6.0%)	
Secondary education	18 (22.5%)	40 (24.0%)	
Intermediate Vocational Education	43 (53.8%)	81 (48.5%)	
Higher Vocational Education and University	16 (20.0%)	36 (21.6%)	
Current smoker	26 (30.6%)	41 (24.1%)	0.27
Weight (kg)	103.9 ± 13.3	103.6 ± 13.6	0.89
BMI	35.9 ± 3.4	36.1 ± 3.4	0.56
Nulliparous	62 (80.5%)	119 (73.5%)	0.23

Participants who completed the FFQ and /or SQUASH either at baseline, 3 months, or 6 months were included. Data are reported as mean ± SD or number (percentage).

**Table 2:** Differences in dietary intake, physical activity, and QoL in the PCOS group compared with non-PCOS group.

	3 Months				6 Months			
	Baseline-Corrected Model		Fully Corrected Model		Baseline-Corrected Model		Fully Corrected Model	
	Differences (95% CI)	*p*-Value	Differences (95% CI)	*p*-Value	Differences (95% CI)	*p*-Value	Differences (95% CI)	*p*-Value
Energy intake (kcal/day)	−70.2 (−272 to 132)	0.50	−110 (−322 to 103)	0.31	−44.7 (−321 to 231)	0.75	−84.2 (−382 to 214)	0.58
Vegetable intake (g/day)	15.0 (−10.0 to 39.9)	0.24	15.8 (−10.6 to 42.2)	0.24	24.2 (1.3 to 47.1)	0.04	23.7 (−0.1 to 47.5)	0.051
Fruit intake (g/day)	−5.4 (−33.3 to 22.5)	0.70	−3.8 (−32.2 to 24.6)	0.79	−3.1 (−34.4 to 28.2)	0.85	−6.0 (−38.3 to 26.3)	0.71
Sugary drinks (glasses/day)	−0.6 (−1.5 to 0.2)	0.13	−0.6 (−1.4 to 0.2)	0.12	−0.9 (−2.3 to 0.5)	0.20	−0.9 (−2.3 to 0.4)	0.17
Alcoholic beverages (yes) ^&^	1.1 (0.5 to 2.2)	0.87	1.0 (0.5 to 2.3)	0.94	1.0 (0.4 to 2.4)	0.96	0.9 (0.4 to 2.4)	0.95
Savory snacks (handful/week)	−0.6 (−3.7 to 2.6)	0.72	−0.7 (−4.1 to 2.7)	0.70	−1.5 (−4.1 to 1.1)	0.27	−1.3 (−4.2 to 1.6)	0.27
Sweet snacks (portion/week) ^@^	1.0 (−1.0 to 3.3)	0.31	1.1 (−1.1 to 3.2)	0.34	2.1 (−0.9 to 5.0)	0.17	2.5 (−0.8 to 5.7)	0.13
Steps (steps/day)	−495 (−1871 to 880)	0.48	−382 (−1839 to 1075)	0.61	214 (−1552 to 1980)	0.81	279 (−1586 to 2143)	0.77
Total MVPA (min/week)	435 (72 to 798)	0.02	363 (−12 to 737)	0.06	−210 (−507 to 86)	0.16	−262 (−572 to 47)	0.10
Leisure time MVPA (min/week)	221 (−69 to 511)	0.14	178 (−123 to 479)	0.25	−100 (−288 to 88)	0.30	−96 (−300 to 108)	0.35
Commuting MVPA (yes) ^&^	1.0 (0.6 to 1.9)	0.91	1.0 (0.5 to 1.9)	0.98	1.0 (0.4 to 2.2)	0.91	0.9 (0.4 to 2.3)	0.91
Physical Component Score	−2.8 (−5.1 to −0.5)	0.02	−2.4 (−4.8 to −0.06)	0.045	−1.1 (−4.4 to 2.1)	0.49	−1.7 (−4.9 to 1.3)	0.27
Mental Component Score	1.4 (−1.8 to 4.6)	0.38	1.0 (−2.3 to 4.4)	0.56	1.0 (−3.3 to 5.2)	0.66	0.5 (−4.1 to 5.1)	0.83

MVPA: moderate to vigorous physical activity. Differences and 95% CI were analyzed by generalized estimating equations, including all women with at least one report of FFQ and/or SQUASH. As all women had different dietary intakes, physical activity levels, and QoL at baseline, we corrected by default for baseline measures. Baseline-corrected model included age, baseline measures, time, PCOS group, and interaction between time and PCOS group. Fully corrected model included age, baseline measures, education, smoking at baseline, time, PCOS group, and interaction between time and group. Negative values indicate lower values in the PCOS group than non-PCOS group and positive values indicate higher values in the PCOS group than non-PCOS group. ^&^ Presented as OR (95% CI). ^@^ One portion of sweet snacks included two biscuits, or two pieces of chocolate, or five candies, or five pieces of licorice.

**Table 3:** Differences in cardiometabolic outcomes in the PCOS group compared with non-PCOS.

	3 Months				6 Months			
	Baseline-Corrected Model		Fully Corrected Model		Baseline-Corrected Model		Fully Corrected Model	
	Differences (95% CI)	*p*-Value	Differences (95% CI)	*p*-Value	Differences (95% CI)	*p*-value	Differences (95% CI)	*p*-Value
Weight (kg)	−0.2 (−1.8 to 1.4)	0.83	−0.5 (−2.2 to 1.2)	0.59	0.9 (−1.6 to 3.4)	0.47	1.1 (−1.6 to 3.8)	0.43
BMI	−0.2 (−0.8 to 0.4)	0.51	−0.3 (−0.9 to 0.3)	0.35	0.5 (−0.4 to 1.4)	0.29	0.5 (−0.4 to 1.4)	0.29
Waist–hip circumference ratio	−0.02 (−0.04 to 0.01)	0.12	−0.02 (−0.04 to 0.01)	0.15	−0.01 (−0.04 to 0.02)	0.48	−0.02 (−0.04 to 0.01)	0.27
Systolic blood pressure (mmHg)	−1.1 (−7.2 to 5.0)	0.72	−0.9 (−7.1 to 5.3)	0.78	−7.4 (−15.3 to 0.5)	0.07	−7.2 (−15.4 to 1.0)	0.08
Diastolic blood pressure (mmHg)	0.9 (−2.7 to 4.6)	0.62	1.3 (−2.5 to 5.0)	0.50	−5.5 (−10.4 to −0.7)	0.02	−5.2 (−10.3 to −0.2)	0.04
Triglycerides (mmol/L)	0.004 (−0.22 to 0.001)	0.97	−0.03 (−0.3 to 0.3)	0.82	−0.1 (−0.3 to 0.2)	0.70	−0.1 (−0.3 to 0.2)	0.67
Total cholesterol (mmol/L)	−0.01 (−0.2 to 0.2)	0.96	−0.04 (−0.3 to 0.2)	0.72	−0.2 (−0.5 to 0.1)	0.25	−0.1 (−0.3 to 0.2)	0.67
HDL-C (mmol/L)	−0.01 (−0.1 to 0.1)	0.67	−0.01 (−0.1 to 0.1)	0.78	−0.04 (−0.1 to 0.05)	0.38	−0.03 (−0.1 to 0.1)	0.57
LDL-C (mmol/L)	−0.1 (−0.2 to 0.1)	0.51	−0.1 (−0.3 to 0.1)	0.39	−0.2 (−0.4 to 0.1)	0.13	−0.1 (−0.3 to 0.1)	0.43
hs-CRP (mg/L)	−0.1 (−1.4 to 1.2)	0.89	−0.2 (−1.5 to 1.2)	0.80	−2.2 (−4.7 to 0.4)	0.09	−2.3 (−5.2 to 0.6)	0.12
Glucose (mmol/L)	0.1 (−0.1 to 0.2)	0.38	0.1 (−0.1 to 0.3)	0.34	0.2 (0.01 to 0.4)	0.04	0.2 (−0.03 to 0.4)	0.09
Insulin (pmol/L)	−15 (−32 to 3.4)	0.11	−16 (−35 to 2.8)	0.09	10 (−9.2 to 30)	0.30	9.0 (−12 to 30)	0.41
HOMA-IR	−0.4 (−1.1 to 0.3)	0.29	−0.5 (−1.2 to 0.3)	0.26	0.5 (−0.2 to 1.2)	0.16	0.5 (−0.3 to 1.2)	0.26
Metabolic syndrome (yes) ^&^	0.53 (0.22 to 1.31)	0.17	0.59 (0.23 to 1.50)	0.27	0.45 (0.13 to 1.55)	0.21	0.48 (0.13 to 1.77)	0.27

HDL-C: high-density lipoprotein cholesterol; LDL-C: low-density lipoprotein cholesterol; hs-CRP: high-sensitivity C-reactive protein; HOMA-IR: homeostasis model assessment of insulin resistance. Differences and 95% CI were analyzed by generalized estimating equations, including all women with at least one report of FFQ and/or SQUASH. As all women had different BMI and cardiometabolic parameters, we corrected by default for baseline measures. Baseline-corrected model included age, baseline measures, time, PCOS group, and interaction between time and PCOS group. Fully corrected model included age, baseline measures, education, smoking at baseline, time, PCOS group, and interaction between time and group. Negative values indicate lower values in the PCOS group than non-PCOS group and positive values indicate higher values in the PCOS group than non-PCOS group. ^&^ Presented as OR (95% CI).

## Data Availability

The data presented in this study are available on request from the corresponding author. The data are not publicly available due to privacy.

## Supplementary Materials

The following are available online at https://www.mdpi.com/article/10.3390/nu13103425/s1, Supplementary Table S1: Dietary intake, physical activity, and QoL at baseline, three months, and six months, Table S2. Cardiometabolic outcomes at baseline, three and six months.
