# Supplementary material for: Effectiveness of a 6-Month Lifestyle Intervention on Diet, Physical Activity, Quality of Life, and Markers of Cardiometabolic Health in Women with PCOS and Obesity and Non-PCOS Obese Controls: One Size Fits All?
Source: Nutrients. 2021 Sep 28;13(10):3425. doi: 10.3390/nu13103425 (PMC8538637; doi:10.3390/nu13103425)
Supplement: Supplementary file 1 [file nutrients-13-03425-s001.zip › nutrients-1394831-supplementary.pdf]

Table S1. Dietary intake, physical activity, and QoL at baseline, three months, and six months.

	PCOS					Non-PCOS				
	**Baseline (N = 84)**	**Three months (N = 50)**	**p-value**	**Six months (N = 33)**	**p-value**	**Baseline (N = 166)**	**Three months (N = 101)**	**p-value**	**Six months (N = 56)**	**p-value**
Energy intake (kcal/day)	1874 (70.0)	1378 (47.0)	<0.001	1415 (46.6)	<0.001	1883 (40.1)	1451 (34.1)	<0.001	1474 (51.0)	<0.001
Vegetable intake (g/day)	124 (6.0)	149 (11.4)	0.03	147 (10.6)	0.01	133 (5.6)	143 (7.0)	0.09	133 (7.5)	0.98
Fruit intake (g/day)	118 (8.8)	130 (11.4)	0.33	123 (11.6)	0.66	111 (6.7)	129 (7.2)	0.003	115 (8.4)	0.60
Sugary drinks (glasses/day)	2.6 (0.4)	1.2 (0.2)	<0.001	1.1 (0.3)	0.001	1.5 (0.2)	0.7 (0.1)	<0.001	0.9 (0.2)	0.01
Alcoholic beverages (glasses/day)	0.1 (0.03)	0.04 (0.01)	0.04	0.04 (0.03)	0.09	0.2 (0.04)	0.1 (0.02)	0.002	0.1 (0.03)	0.01
Savory snacks (handful/week)	7.5 (1.4)	2.7 (0.5)	0.001	2.5 (0.5)	<0.001	6.4 (0.6)	2.1 (0.3)	<0.001	3.1 (0.4)	<0.001
Sweet snacks (portion/week)[@]	5.9 (1.0)	2.5 (0.5)	0.001	3.4 (0.9)	0.05	6.2 (0.7)	2.4 (0.4)	<0.001	2.2 (0.4)	<0.001
Steps (steps/day)	5962 (393.6)	8790 (658.7)	<0.001	9264 (468.5)	<0.001	6086 (286.8)	9160 (372.0)	<0.001	9208 (569.2)	<0.001
Total MVPA (min/week)	567 (73.8)	1059 (151.8)	0.001	532 (105.3)	0.77	648 (58.7)	772 (99.1)	0.24	820 (107)	0.09
Leisure time MVPA (min/week)	275 (46.8)	541 (107)	0.01	217 (40.9)	0.34	344 (45.7)	415 (81.0)	0.44	394 (69.2)	0.45
Commuting MVPA (min/week)	53.6 (11.3)	59.4 (17.3)	0.72	61.3 (20.7)	0.71	59.3 (11.7)	70.8 (17.6)	0.45	70.0 (15.4)	0.41
Physical Component Score	49.0 (1.1)	50.3 (1.3)	0.13	51.0 (1.4)	0.09	49.5 (0.8)	52.7 (0.8)	<0.001	52.7 (1.0)	0.003
Mental Component Score	49.9 (0.9)	48.9 (1.4)	0.43	50.0 (1.4)	0.95	50.5 (0.8)	49.6 (1.0)	0.44	49.3 (1.4)	0.40

MVPA: moderate to vigorous physical activity. Data are presented as estimated marginal means (SE) from generalized estimating equations analyses. p-values indicates the difference between three months and baseline or between six months and baseline in PCOS with PCOS and non-PCOS women, respectively. [@] One portion of sweet snacks included 2 biscuits, or 2 pieces of chocolate, or 5 candies, or 5 pieces of liquorice.

Table S2. Cardiometabolic outcomes at baseline, three and six months.

	PCOS					Non-PCOS				
	**Baseline (N = 84)**	**Three months (N = 50)**	**p-value**	**Six months (N = 33)**	**p-value**	**Baseline (N = 166)**	**Three months (N = 101)**	**p-value**	**Six months (N = 56)**	**p-value**
Weight (kg)	104 (1.4)	100 (1.6)	<0.001	100 (1.6)	<0.001	104 (1.0)	100 (1.1)	<0.001	98.7 (1.3)	<0.001
BMI	35.9 (0.4)	34.4 (0.4)	<0.001	34.5 (0.5)	<0.001	36.1 (0.3)	34.9 (0.3)	<0.001	34.4 (0.4)	<0.001
Waist circumference (cm)	108 (1.0)	104 (1.3)	<0.001	103.5 (1.2)	<0.001	108 (0.7)	105 (0.9)	<0.001	104 (1.0)	<0.001
Hip circumference (cm)	124 (0.9)	122 (1.2)	0.02	120 (1.2)	<0.001	126 (0.7)	122 (0.8)	<0.001	120 (1.0)	<0.001
Waist-hip circumference ratio	0.88 (0.009)	0.86 (0.01)	0.04	0.86 (0.01)	0.22	0.86 (0.004)	0.86 (0.006)	0.78	0.86 (0.007)	0.78
Systolic blood pressure (mmHg)	125 (1.7)	125 (2.6)	0.997	114 (2.4)	<0.001	127 (0.9)	128 (1.5)	0.38	125 (2.4)	0.44
Diastolic blood pressure (mmHg)	78.4 (1.0)	80.0 (1.4)	0.31	76.1 (1.4)	0.099	80.6 (0.6)	80.9 (1.2)	0.77	83.2 (1.8)	0.15
Triglycerides (mmol/L)	1.2 (0.1)	1.3 (0.1)	0.53	1.0 (0.1)	0.02	1.3 (0.1)	1.3 (0.1)	0.87	1.2 (0.1)	0.18
Total cholesterol (mmol/L)	4.7 (0.1)	4.6 (0.1)	0.38	4.5 (0.1)	0.10	4.8 (0.1)	4.7 (0.1)	0.05	4.8 (0.1)	0.76
HDL-C (mmol/L)	1.1 (0.03)	1.1 (0.03)	0.99	1.2 (0.04)	0.12	1.2 (0.02)	1.2 (0.03)	0.56	1.2 (0.04)	0.007
LDL-C (mmol/L)	3.1 (0.1)	2.9 (0.1)	0.04	2.9 (0.1)	0.02	3.2 (0.1)	3.0 (0.1)	0.02	3.1 (0.1)	0.74
hs-CRP (mg/L)	5.6 (0.5)	5.3 (0.7)	0.57	4.9 (0.7)	0.19	5.4 (0.4)	5.2 (0.4)	0.71	6.7 (1.2)	0.25
Glucose (mmol/L)	5.3 (0.1)	5.3 (0.1)	0.63	5.4 (0.1)	0.36	5.3 (0.04)	5.3 (0.1)	0.39	5.2 (0.1)	0.22
Insulin (pmol/L)	115 (7.4)	92.3 (7.5)	0.004	116 (9.8)	0.97	90.2 (3.6)	83.3 (4.1)	0.04	78.3 (4.7)	0.005
HOMA-IR	4.0 (0.3)	3.2 (0.3)	0.02	4.1 (0.4)	0.81	3.1 (0.1)	2.8 (0.2)	0.06	2.7 (0.2)	0.01
Metabolic syndrome (yes)[&]	40/65 (61.5%)	12/32 (37.5%)	0.03	5/16 (31.3%)	0.04	70/142 (49.3%)	31/65 (47.7%)	0.55	15/34 (44.1%)	0.40

HDL-C: high-density lipoprotein cholesterol; LDL-C: low-density lipoprotein cholesterol; hs-CRP: high-sensitivity C-reactive protein; HOMA-IR: homeostasis model assessment of insulin resistance. Data are presented as estimated marginal means (SE) from generalized estimating equations analyses. [&] Presented as number (percentage). p-values indicates the difference between three months and baseline or between six months and baseline in women with PCOS and non-PCOS women, respectively.
